# Maternal Coronavirus Infections and Neonates Born to Mothers with SARS-CoV-2: A Systematic Review

**DOI:** 10.3390/healthcare8040511

**Published:** 2020-11-24

**Authors:** Waldemar Naves do Amaral, Carolina Leão de Moraes, Ana Paula dos Santos Rodrigues, Matias Noll, Jalsi Tacon Arruda, Carolina Rodrigues Mendonça

**Affiliations:** 1 Programa de Pós-Graduação em Ciências da Saúde, Universidade Federal de Goiás, 74605-050 Goiânia, Brazil; waldemar@sbus.org.br (W.N.d.A.); carolina.leao.moraes2@gmail.com (C.L.d.M.); anapsr@gmail.com (A.P.d.S.R.); 2 Instituto Federal Goiano, Campus Ceres, 76300-000 Ceres, Brazil; matias.noll@ifgoiano.edu.br; 3 Departamento de Medicina, Centro Universitário de Anápolis—UniEvangélica, 75083-515 Anápolis, Brazil; jalsitacon@gmail.com

**Keywords:** pregnant woman, coronavirus, infectious disease transmission, vertical transmission, obstetric management, SARS-CoV-2, systematic review

## Abstract

The coronavirus disease 2019 (COVID-19) pandemic is continuously affecting the lives of all people. Understanding the impact of COVID-19 on pregnancy in terms of morbidity, mortality, and perinatal maternal and fetal outcomes is essential to propose strategies for prevention and infection control. Here, we conducted a systematic review to investigate pregnant women infected with COVID-19 in terms of signs and symptoms, type of delivery, comorbidities, maternal and neonatal outcomes, and the possibility of vertical transmission. A search on Embase and PubMed databases was performed on 31 October 2020. Observational studies and case reports on pregnant women infected with COVID-19 were included without language restrictions. The 70 selected studies included a total of 1457 pregnant women diagnosed with COVID-19 in the first, second, and third trimesters of pregnancy. The most common signs and symptoms were fever, cough, and nausea. The most frequent comorbidities were obesity, hypertensive disorders, and gestational diabetes. Among maternal and fetal outcomes, premature birth (*n* = 64), maternal death (*n* = 15), intrauterine fetal death or neonatal death (*n* = 16), cases of intrauterine fetal distress (*n* = 28), miscarriage (*n* = 7), decreased fetal movements (*n* = 19), and severe neonatal asphyxia (*n* = 5) were the most frequent. Thirty-nine newborns tested positive for SARS-CoV-2. Additionally, severe acute respiratory syndrome coronavirus 2 (SARS-CoV-2) RNA was detected in the placenta (*n* = 13) and breast milk (*n* = 6). This review indicates that COVID-19 during pregnancy can result in maternal, fetal, and neonatal complications. In addition, SARS-CoV-2 viral exposure of neonates during pregnancy and delivery cannot be ruled out. Thus, we highlight the need for long-term follow-up of newborns from mothers diagnosed with COVID-19 to establish the full implications of SARS-CoV-2 infection in these children.

## 1. Introduction

Coronavirus disease 2019 (COVID-19) is an infectious condition caused by severe acute respiratory syndrome coronavirus 2 (SARS-CoV-2). It was first reported in December 2019 after an outbreak of pneumonia of unknown etiology was identified in Wuhan, China [1,2]. Currently, the virus continues to spread to different regions of the world, including several countries in Europe and the United States, which reported the highest number of confirmed cases and deaths in March and April [2].

With continuous emergence of new data, there is an increasing understanding of the mechanisms of the disease [3]. Although studies about the effects of COVID-19 on pregnancy are expanding, there are still many unanswered questions [4,5]. Data regarding COVID-19 and its effects on both mother and fetus or newborn are still scarce, and the potential risk of vertical transmission is a major concern [6]. It is well established that pregnant women, in general, are vulnerable to infections; therefore, both pregnant women and newborns should be considered at risk for COVID-19 [6,7]. Thus, it is important to understand the impact of COVID-19 on pregnant women [6,8] in terms of morbidity, mortality, and perinatal maternal and fetal outcomes [7,9] to propose strategies for prevention and infection control [6].

Systematic reviews on the topic have already been published [10,11,12,13] and indicated that neonatal COVID-19 infection is low, and uncommonly symptomatic. As COVID-19 infection is growing in different cities around the world, new research is being published all the time. In this sense, a broad and current research on the maternal clinical characteristics of the COVID-19 infection and the neonatal results, during childbirth or postnatal (by environmental exposure), can provide important new information to guide clinical and preventive practice guidelines. Therefore, we aimed to investigate pregnant women infected with COVID-19 in terms of signs and symptoms, type of delivery, comorbidities, clinical outcomes (maternal and neonatal), and possibility of vertical transmission (via placenta or hematogenous route, birth canal, and lactation) through a systematic review. We believe that these findings will make a significant contribution to the current clinical and preventive practice guidelines worldwide.

## 2. Materials and Methods

A systematic review on pregnancy and COVID-19 was performed according to the Preferred Reporting Items for Systematic Reviews and Meta-Analyses (PRISMA) [14] guideline. Our study was conducted in six stages: (1) Formulation of the study question, (2) elaboration of inclusion and exclusion criteria, (3) definition of the information to be extracted from the identified and selected articles, (4) analysis, (5) interpretation of results, and (6) presentation of the review [15]. The protocol was registered with PROSPERO (CRD42020220263). 

### 2.1. Information Sources and Search Strategy

An electronic search was performed in Embase and PubMed databases for articles published until 31 October 2020. The reference lists of selected articles and information available on Google Scholar were also searched. The following keywords were used for the searches: Pregnancy, pregnant woman, pregnant women, COVID-19, SARS-CoV-2, and vertical transmission. Operator fields were filled out with AND/OR. We used the following terms to search in PubMed: ((Pregnancy) OR (pregnant woman) OR (pregnant women)) AND ((COVID-19) OR (SARS-CoV-2) OR (coronavirus pregnancy) AND (vertical transmission)).

### 2.2. Eligibility Criteria

The inclusion criteria were as follows: (1) Outpatient or population-based observational studies (prospective or retrospective) or case reports and pre-print articles (2) in any language, (3) published between December 2019 and October 2020; (4) studies with pregnant women with laboratory diagnosis for COVID-19; and (5) pregnant women in any gestational trimester. Letters to the editor, opinions, comments, correspondence articles reporting previously published data, reviews, guidelines, and duplicate studies (i.e., found in more than one database) were excluded.

### 2.3. Data Extraction and Quality Assessment 

**Figure 1:**
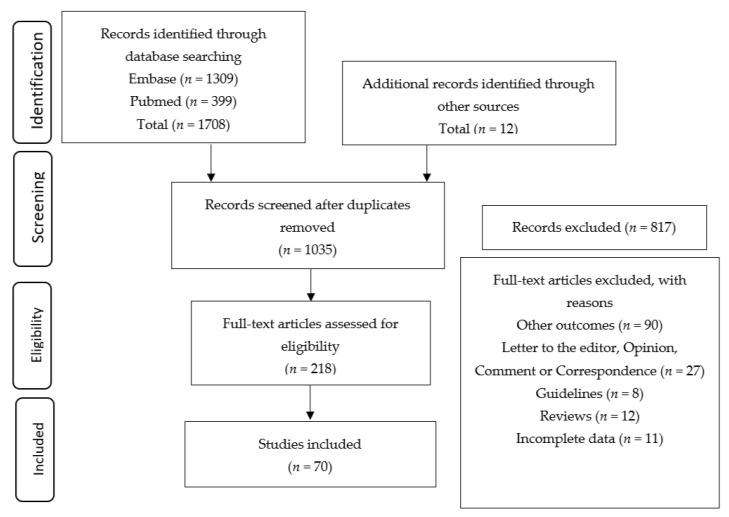
Preferred Reporting Items for Systematic Reviews and Meta-Analyses (PRISMA) flow diagram.

Titles and abstracts were used to screen for potentially eligible studies. The identified studies were then read in full and critically evaluated by three members of the research team (C.R.M., C.L.M., and J.T.A.) (Figure 1) based on their knowledge on pregnancy and COVID-19. Doubts and/or disagreements about the articles were discussed by the research team to make a consensus decision. The data extracted from the studies were as follows: (1) Signs and symptoms of the mother and fetus, (2) gestational age and pregnancy results (maternal or fetal death), (3) type of delivery (natural, emergency or elective cesarean section, abortion, or complications), and (4) possibility of SARS-CoV-2 vertical transmission.

The quality of the studies was assessed using the Grading of Recommendations, Assessment, Development, and Evaluations (GRADE) [16,17]. The quality of the evidence from the studies was classified into four categories: High, moderate, low, or very low [16,17]. 

## 3. Results

A flow diagram based on the PRISMA guideline was created to represent the different stages of article selection (Figure 1).

### 3.1. Study Selection

From the 1720 studies retrieved, 1708 were identified in Embase and PubMed while 12 were taken from other sources. After exclusion of duplicate studies, a total of 1035 titles and abstracts were collected. Among these, 218 manuscripts were retrieved for full reading by the three authors (C.R.M., C.L.M., and J.T.A.) independently. A total of 70 studies met the inclusion criteria [5,7,9,18,19,20,21,22,23,24,25,26,27,28,29,30,31,32,33,34,35,36,37,38,39,40,41,42,43,44,45,46,47,48,49,50,51,52,53,54,55,56,57,58,59,60,61,62,63,64,65,66,67,68,69,70,71,72,73,74,75,76,77,78,79,80,81,82,83,84,85]. The main findings of the selected studies are shown in Table 1.

The distribution of studies in terms of quality of evidence based on GRADE are as follows: High quality (*n* = 1) [41], moderate quality (*n* = 11) [9,21,22,33,48,49,50,52,55,63,68], low quality (*n* = 26) [5,9,23,24,25,26,28,29,36,40,43,46,47,51,54,56,60,61,62,64,65,66,67,69,70,73], and very low quality (*n* = 32) [7,18,20,27,30,31,32,34,35,37,38,39,42,44,45,53,57,58,59,66,71,72,74,75,77,78,79,80,81,83,84,85]. The studies that were classified as “low-quality” and “very low-quality” are case reports or small series of cases which also characterizes a high risk of bias.

### 3.2. Synthesis of Results 

Of the 70 studies included, 34 were carried out in China [5,9,25,26,27,28,30,31,32,42,43,44,45,46,47,53,54,57,58,59,60,61,62,63,64,65,66,67,68,69,70,73,78], 10 in the United States [21,22,23,37,48,49,52,75,79,85], eight in Italy [24,33,51,55,81,82,83,84], three in Iran [36,39,76], one in Korea [7], one in Turkey [38], one in Peru [18], one in Switzerland [20], two in France [56,74], three in Spain [50,77,80], one in Australia [71], one in Spain [72], one in the Netherlands and Ireland [34], one in Canada [40], one in the United Kingdom [41], and one in Sweden [35].

A total of 1457 pregnant women diagnosed with COVID-19 and 1042 newborns from infected mothers were included. Maternal SARS-CoV-2 infection was diagnosed by reverse transcriptase–polymerase chain reaction (RT-PCR or PCR) of nasopharyngeal swabs and sputum samples [9].

### 3.3. Signs and Symptoms

Among infected pregnant women, 116 (7.9%) were asymptomatic at the beginning of medical care. Of the symptomatic pregnant women, the most frequent symptoms were fever (>37.3 °C) (*n* = 695, 47.7%), cough (*n* = 647, 44.4%), and nausea (*n* = 148, 10.2%). Less common symptoms included dyspnea (*n* = 87, 6.5%), fatigue (*n* = 58, 4.3%), myalgia (*n* = 42, 2.9%), and diarrhea (*n* = 14, 0.9%). In the immediate postpartum period, the most frequent symptom was fever (37.8–39.33 °C).

### 3.4. Gestational Age

Twenty-one (1.4%) of the pregnant women were in the first trimester, 97 (6.6%) in the second trimester, and 1339 (91.9%) in the third trimester of pregnancy.

### 3.5. Type of Delivery

In terms of the type of delivery, 597 (57.3%) underwent elective cesarean section, 36 (3.4%) received emergency cesarean sections, and 364 (34.9%) went through spontaneous vaginal delivery.

### 3.6. Comorbidity and Pregnancy Complications

The most reported maternal comorbidities were obesity (*n* = 191, 13.1%), hypertensive disorders (*n* = 117, 8.0%), diabetes (*n* = 49, 3.3%), asthma (*n* = 44, 3.0%), and preeclampsia (*n* = 15, 1.0%). Pregnancy complications included gestational diabetes (*n* = 91, 6.2%) and gestational hypertension (*n* = 12, 0.8%). Comorbidities and complications in pregnant women with COVID-19 are described in Table 2.

### 3.7. Maternal Outcomes

Among pregnant women diagnosed with COVID-19, 68 (4.6%) were admitted in intensive care units (ICU) [21,22,23,33,36,55,56,67,85]. There were 15 (1.0%) cases of maternal death [21,36,39,41], mostly from United Kingdom (*n* = 5) [41] and Iran (*n* = 7) [36]. Hantoushzadeh et al. [36] reported that pregnant women with SARS-CoV-2 infection in the second or third trimester of pregnancy can suffer cardiopulmonary complications and die.

### 3.8. Neonatal and Fetal Outcomes

Among neonates born to infected mothers, 187 (17.9%) required admission to neonatal ICUs [18,26,33,41,50,55,56,63,65,66,85]. There were 16 (1.5%) total cases of neonatal death and fetal intrauterine death [20,36,39,41,42,48,49,63,70]. Karami et al. reported a case of an infected pregnant woman who vaginally delivered a cyanotic fetus in the third trimester [39]. In the study by Zhu et al. [67], a newborn delivered at 34 + 5 weeks of gestation from a 30-year-old mother with COVID-19 experienced refractory shock, gastric bleeding, multiple organ failure, and disseminated intravascular coagulation. There were four total cases of fetal demise: One at 17 weeks [49] and three at the third trimester of pregnancy [42,48,63]. Lookken et al. [48] reported one case of stillbirth at 38.7 weeks in which the qualitative PCR tests of placental and fetal tissue were negative for SARS-CoV-2 and cytomegalovirus. However, the delay between fetal death and sample extraction for PCR analysis may have led to inaccurate results. 

In the study by Li et al. [42], biochemical examination of umbilical cord blood at birth revealed a marked increase in myocardial enzymes, suggesting severe damage of the fetal myocardium. Considering severe hypoxia, the possibility of immunologic damage cannot be ruled out. This may have led to difficulties in resuscitation and eventually neonatal death. Maternal hypoxia and unstable circulation secondary to COVID-19 can endanger the fetus and cause intrauterine fetal death [42].

Among the seven total cases (0.7 %) of miscarriage [20,24,41,56], the distribution by type are as follows: Spontaneous miscarriage (*n* = 1) [24], threatened miscarriage (*n* = 1) [29], medical miscarriage (*n* = 4) [54], and induced miscarriage (*n* = 1) [29].

Among various studies, there were 64 (6.1%) cases of premature birth [25,36,54,61,63,64,69,70,73,75,76,77,82,85], 10 (0.9%) patients with complications in pregnancy [73], and 28 (2.7%) cases of intrauterine fetal distress [25,47,48,53,60,63,68,69,70,73,84]. 

Decreased fetal movements were reported for 19 (1.8%) fetuses [21,30,36,37,38,49,57,58,68,69]. There were five (0.5%) cases of severe neonatal asphyxia [63,64,69] and four (0.3%) cases of low birth weight (<2500 g) in the third pregnancy trimester [25,63]. Abnormal fetal heart monitoring [29], fetal tachycardia [28,34], and placental detachment [48] were also reported. Other outcomes include premature rupture of membranes (*n* = 26, 2.5%) [36,50,62,69,86], abnormal amniotic fluid (*n* = 3, 0.3%) [69,70], and abnormal umbilical cord in the third pregnancy trimester (*n* = 6, 0.6%) [64,70].

### 3.9. Newborns and Placental and Breast Milk Samples Tested Positive for SARS-COV-2

Of the 70 studies analyzed, 21 studies included a total of 39 (3.7%) newborns who tested positive for SARS-CoV-2 [18,24,33,36,40,41,50,51,55,58,66,68,74,75,76,82,83]. In only five studies (23.8%), newborns were tested within the first 12 h of birth [33,41,50,74,76]. Two studies have presented neonates with symptoms; however, tests for SARS-CoV-2 were negative [32,35]. SARS-CoV-2 RNA was detected in 13 placenta samples [20,51,52,74,79,81,82,83,85] and six breast milk samples of infected pregnant women [61,82,84]. There was also a positive test for SARS-CoV-2 RT-PCR in umbilical cord and vagina samples [82] (Table 3). In addition, one newborn received an inconclusive result but was otherwise asymptomatic [22]. Twelve newborns presented IgG positive in umbilical cord plasma [82] and two neonates born to a mother with COVID-19 had elevated antibody levels (IgM) 2 h after birth [31,82].

### 3.10. Newborns Tested Negative for SARS-COV-2 and Vertical Transmission

A total of 959 newborns were asymptomatic at birth and had negative results for SARS-CoV-2. The distribution of oropharyngeal swab collection time for RT-PCR among studies in which newborns were negative are as follows: At birth (*n* = 51) [21,24,31,32,33,34,35,36,38,44,47,48,49,51,54,56,58,61,70,71,73,77,78,80], 72 h after birth (*n* = 1) [70], fifth day (*n* = 1) [69], seventh and ninth days (*n* = 1) [70], and fourth to fourteenth days (*n* = 1) [46]. 

In addition to oropharyngeal swabs, other samples for testing included placental tissue [7,26,27,29,30,32,38,44,47,53,58,59,62,78], fetal membrane [27], umbilical cord blood [5,7,20,29,32,38,44,45,53,58,59,65,78], breast milk [25,29,31,32,35,38,44,45,47,53,57,58,80], amniotic fluid [7,20,25,29,44,45,53,59,62,65,78], serum [32,53], bronchoalveolar lavage fluid [53], vaginal secretions [20,31,32,53], axillary swab [20], mouth swab [20], neonatal gastric fluid [78], meconium [20], urine [44,45,53], rectal swab samples [24,56], feces [44,45,57], and anal swab [53,61,78]. In addition to RT-PCR, other tests such as IgM and IgG antibody [31,64], cytokine [31], and blood biochemistry tests [31] were also used. The results of all the various tests using a myriad of samples were negative.

In one study, the presence of SARS-CoV-2 was investigated in vaginal discharge and amniotic fluid in four pregnant women with mild acute symptoms of COVID-19 who underwent amniocentesis during the second trimester of pregnancy [72]. In addition, in another study, a case of vaginal delivery without complications was described in a mother with COVID-19 [47]. The test for neonatal COVID-19 24 h after delivery was still negative despite the fact that the infant was breastfed and not separated from the mother [71].

## 4. Discussion

This systematic review gathered evidence available on pregnancy and SARS-CoV-2 infection from the international literature to investigate signs and symptoms, type of delivery, comorbidities, clinical outcomes (maternal and neonatal), and vertical transmission risk of COVID-19. This study included a significant number of newborns and pregnant women diagnosed with COVID-19 in the first, second, and third trimesters of pregnancy. Although most studies which were included were case reports or case series that have low levels of evidence quality, these are still important in the current context due to the need for information to support public health policies. 

In terms of signs and symptoms of COVID-19, fever above 37.0 °C was the most frequent symptom reported by pregnant women, followed by cough and nausea in the prenatal period. In the postpartum period, subjects were reported to experience fever (37.8–39.33 °C) and a general worsening of the condition, especially in those who were initially asymptomatic. Among symptomatic pregnant women, 4.99% were admitted in the ICU. The results in our study are consistent with those of studies in the general population, where fever and coughing were the most reported symptoms [87]. 

Obesity and hypertensive disorders were the most reported comorbidities in pregnant women with COVID-19. It is noted that the majority of the pregnant women did not have serious complications, with a low occurrence of maternal death (1.0%) and premature rupture of the membrane (2.5%). However, pregnancy complications, including gestational diabetes and gestational hypertension, were reported. Many pregnant women presented with worsening of the general condition that required an induced delivery or emergency cesarean section. However, we found that the maternal risk in pregnant women diagnosed with COVID-19 was relatively low. 

In contrast, 17.9% of newborns were admitted in the neonatal ICU. Fetal and neonatal complications including premature delivery (6.1%), fetal distress (2.7%), decreased fetal movements (1.8%), and fetal and neonatal death (1.5%) were identified. There were also cases of miscarriage and severe neonatal asphyxia. Therefore, we hypothesize that there is a greater risk of fetal and neonatal complications in the first and second trimesters of pregnancy.

This systematic review included cases where SARS-CoV-2 RNA was detected in the placenta (*n* = 13), breast milk (*n* = 6), and neonates (*n* = 39). It is important to note that only five studies confirmed a diagnosis within the first 12 h of birth. In other studies, SARS-CoV-2 was detected 12 h after birth, hinting at the possibility of late-onset neonatal infection. Nevertheless, we highlight that 92% of newborns from mothers infected with COVID-19 did not acquire the infection during birth. 

Although there is a theoretical risk of vertical transmission, it seems to be low and, so far, remains poorly understood. It has been reported that the placental barrier does not allow for the passage of SARS-CoV-2 [88]. However, there is evidence of histopathologic placental changes in women infected with COVID-19, showing poor maternal vascular perfusion and inflammation [89]. It is not clear whether this can disrupt the maternal–placental interface to allow the transplacental transmission of SARS-CoV-2 [89]. Furthermore, its role in the occurrence of premature births and other fetal complications remains unknown. Thus, more robust studies, preferably longitudinal studies, involving a large sample size with long-term follow-up are crucial to establish the full implications of COVID-19 on pregnancy and early development. It is unclear whether maternal and newborn COVID-19 infection will cause any sequelae in childhood. Nevertheless, it is essential to formulate guidelines for the management of pregnant women infected with SARS-CoV-2 as a way to minimize viral exposure and transmission [7]. These protocols also play important roles in protecting the medical team and providing a suitable hospital environment (e.g., respiratory precautions, use of personal protective equipment, and negative pressure rooms) [90].

The Lancet Infectious Diseases has published guidelines on the management of pregnant women exposed to COVID-19. For asymptomatic cases, home isolation for 14 days is recommended. In symptomatic cases, key recommendations include prioritization of vaginal delivery when possible, late fixation of the umbilical cord, without early cleaning of the newborn, and isolated neonatal surveillance. In both cases, mother–child separation and breastfeeding are discussed individually by an interdisciplinary team [19]. In addition, a recent guideline published in June recommends that for cases where separation is not applicable, other measures to reduce risk of infection (e.g., physical barriers and face mask) must be adopted. For those who choose to breastfeed, mothers must wear face masks and practice good hygiene (hand and breast) before each feeding. Newborns from mothers with confirmed or suspected COVID-19 at the time of the delivery should be tested 24 h after birth. If negative, another test at approximately 48 h must be done if testing capacity is available [91].

### 4.1. Strengths and Limitations

This review was completed after an extensive bibliographic search using two databases, reference lists, and Google Scholar. We included a large number of pregnant women diagnosed with COVID-19 from 16 countries and data on the first, second, and third trimesters of pregnancy. However, our study has some limitations: First, our findings are mostly limited to case reports and retrospective studies with a small number of cases analyzed. Second, there was a lack of methodological criteria in the conduction of many included studies, which can contribute to erroneous results. However, it is important to highlight that we have gathered all the evidence available in the literature to date and that this information is important to guide health and management policies for pregnant women affected by COVID-19 in the first, second, and third trimesters of pregnancy. In addition, the justification for conducting our study is the need to quickly assess and discuss the evidence that has been generated. Finally, some relevant publications may have been released during the submission or publication process of this paper.

### 4.2. Future Recommendations

With the worsening of the COVID-19 global situation, new well-designed research is needed to clarify the risk of vertical transmission (via placenta or hematogenous routes, birth canal, and lactation) of SARS-CoV-2. In addition, further studies are necessary to investigate potential therapeutic interventions that prevent maternal and neonatal morbidity and possible sequelae resulting from COVID-19 infection. In addition, it is important that future studies assess complications arising from COVID-19 in pregnant women in the first and second trimester. These studies are important to improve clinical and preventive strategies for managing COVID-19 in pregnant women and their newborns. 

## 5. Conclusions

This review revealed that pregnant women with COVID-19 usually present with fever, cough, and nausea. Among various comorbidities, obesity and hypertensive disorders are the most common. It is important to highlight the prevalence of premature birth, maternal death, premature rupture of the membrane, intrauterine fetal death, neonatal death, miscarriage, decreased fetal movements, and severe neonatal asphyxia among cases of infected mothers. Although we found only 27 cases of newborns infected with COVID-19, viral exposure of SARS-CoV-2 during pregnancy and intrapartum period cannot be ruled out and should be further investigated in future studies. Thus, it is important to follow-up all newborns from mothers diagnosed with COVID-19.

## Figures and Tables

**Table 1:** Data on signs and symptoms, gestational age, type of delivery, comorbidity, and vertical transmission of coronavirus disease 2019 (COVID-19) in pregnant women.

First Author, Year, and Country	Study Size and Age	Study Design	Signs and Symptoms		Gestational Age	Type of Delivery and Maternal or Fetal Complications	GRADE *
			**Before Delivery**	**Postpartum**			
Algarroba, et al., 2020 [85] EUA	*n* = 1 Age: 40 years	Case report	Worsening shortness of breath, cough, and hypoxia	NR	Third trimester (28 weeks gestational age)	Cesarean delivery (*n* = 1)	●○○○
Alzamora et al., 2020 [18] Peru	*n* = 1 Age: 41 years	Case report	General malaise, fatigue, and low-grade fever	NR	Third trimester (33 weeks gestational age)	Emergency cesarean section (*n* = 1)	●○○○
Baud et al., 2020 [20] Switzerland	*n* = 1 Age: 28 years	Case report	Fever (39.2 °C), myalgia, fatigue, mild pain with swallowing, diarrhea, and dry cough for 2 days	NR	Second trimester (19 weeks gestational age)	Vaginal deliveries (after 10 h of labor) (*n* = 1)	●○○○
Blitz et al., 2020 [21] USA	*n* = 13 Mean maternal age 33.8 ± 5.2	Case series	Fever, subjective or measured (*n* = 12) Cough (*n* = 13) Dyspnea (*n* = 10) Myalgia (*n* = 6) Fatigue or malaise (*n* = 3)	NR	Third trimester (mean weeks gestational age 33.3 ± 5.3)	Cesarean for acute respiratory decompensation (*n* = 5) Cesarean for obstetrical indication (*n* = 1) Vaginal delivery (*n* = 1)	●●●○
Breslin et al., 2020 [22] USA	*n* = 43 Mean maternal age 29.7 ± 6.0	Case series, retrospective	Symptomatic gestates (*n* = 29) Asymptomatic pregnant women (*n* = 14) Fever ≥37.5 °C (100.0 °F) (*n* = 14) Cough (*n* = 19) Myalgia or fatigue (*n* = 11) Dyspnea (*n* = 7) Headache (*n* = 8) Chest pain (*n* = 5)	Worse fever or increased breathing work	Third trimester (≥37 weeks gestational age)	Cesarean delivery (*n* = 8) Vaginal deliveries (*n* = 18)	●●●○
Breslin et al., 2020 [23] USA	*n* = 7 Age: 27 to 39 years	Case series	Fever ≥37.0 °C (100.0 °F) (*n* = 2) Cough (*n* = 3) Myalgias (*n* = 3) Chest pain (*n* = 2) Headache (*n* = 2)	Fever Severe hypertension Cough Severe bronchospasm and disproportionate reactive lung disease	Third trimester (≥37 weeks gestational age)	Emergency cesarean section (*n* = 7)	●●○○
Buonsenso et al., 2020 [24] Italy	*n* = 7 Age: 27 to 39 years	Observational study	NR Asymptomatic (*n* = 1)	NR	Second trimester (≥20 weeks gestational age)	Cesarean section (*n* = 2)—third trimester	●●○○
Chen et al., 2020 [28] China	*n* = 5 Age: 25 to 31 years	Descriptive study	Cough Sputum Coryza Asymptomatic (*n* = 3)	Low fever (37.5–38.5 °C) (*n* = 5)	Third trimester (39–40 weeks)	Emergency cesarean section (*n* = 1) (preeclampsia—fetal tachycardia) Elective cesarean section (*n* = 1) (gestational diabetes) Vaginal delivery (*n* = 3)	●●○○
Chen et al., 2020 [25] China	*n* = 9 Age: 26 to 40 years	Descriptive, retrospective study	Low fever without chills (*n* = 7) Myalgia (*n* = 3) Malaise (*n* = 2) Cough (*n* = 4) Dyspnea (*n* = 1) Sore throat (*n* = 2) Diarrhea (*n* = 1)	Fever (variation of 37.8–39.33 °C) (*n* = 6)	Third trimester (≥36 weeks gestational age)	Cesarean (*n* = 9)	●●○○
Chen et al., 2020 [27] China	*n* = 3 Age: 23 to 32 years	Descriptive, retrospective study	Fever and chest tightness (*n* = 1)	Fever (*n* = 3)	Third trimester (≥35 weeks gestational age)	Emergency cesarean section (*n* = 3)	●○○○
Chen et al., 2020 [26] China	*n* = 17 Mean maternal age 29.7 years	Descriptive, retrospective study	Mild fever without chills (≤39 °C) (*n* = 4) Cough (*n* = 4) Fatigue (*n* = 1) Chest distress (*n* = 2) Dyspnea (*n* = 1) Diarrhea (*n* = 1)	NR	Third trimester (≥35 weeks gestational age)	Elective cesarean delivery (*n* = 14) Emergency cesarean delivery (*n* = 3)	●●○○
Chen et al., 2020 [30] China	*n* = 4 Age: 23 to 34 years	Case report	Fever (*n* = 3) Cough (*n* = 2) Fatigue (*n* = 2) Headache (*n* = 2) Dyspnea (*n* = 2)	Anemia and dyspnea	Third trimester (≥37 weeks gestational age)	Cesarean section (*n* = 3) Vaginal delivery (*n* = 1)	●○○○
Chen et al., 2020 [29] China	*n* = 3 Age: 23 to 34 years	Case report	Cough (*n* = 3) Fever (*n* = 3) Fatigue (*n* = 3) Diarrhea, nausea, and vomiting	NR	First trimester (6 weeks) Second trimester (25 weeks) Third trimester (35 weeks)	Cesarean section delivery (*n* = 1)	●●○○
Costa et al., 2020 [84] Italy	*n* = 2 Age: 38 years and 42 years	Case report	Fever, shortness of breath, and diarrhea (*n* = 1) Cough (*n* = 1)	NR	Third trimester (*n* = 2) (≥34 weeks gestational age)	Caesarean section (*n* = 2)	●○○○
Dong et al., 2020 [31] China	*n* = 1 Age: 29 years	Case report	Fever, nasal congestion Liver injury	NR	Third trimester (34 weeks)	Cesarean section delivery (*n* = 1)	●○○○
Elósegui et al., 2020 [72] Spain	*n* = 4 Age: 27 to 40 years	Case series	Mild clinical symptoms	NR	Second trimester	SARS-CoV-2 in vaginal discharge and amniotic fluid in Caucasian pregnant women affected by mild acute symptoms of COVID-19	●○○○
Facchetti et al., 2020 [83] Italy	*n* = 1 Age: 29	Case report	Fever and idiopathic thrombocytopenia	NR	Third trimester (37 weeks gestational age)	Vaginal delivery was induced (*n* = 1)	●○○○
Fan et al., 2020 [32] China	*n* = 2 Age: 34 years and 29 years	Case report	Nasal congestion (*n* = 2) Fever (37. 3–38.5 °C) (*n* = 2) Skin rash (*n* = 1) Chill (*n* = 1) Sore throat (*n* = 1)	NR	Third trimester (37 weeks gestational age) (36 weeks gestational age)	Cesarean section delivery (*n* = 2)	●○○○
Ferrazzi et al., 2020 [33] Italy	*n* = 42 Mean maternal age 32.9	Retrospective multicenter study	Fever (*n* = 20), cough (*n* = 7), myalgia/malaise (*n* = 7), dyspnea (*n* = 8)	NR	Third trimester (±30 weeks gestational age)	Elective cesarean (*n* = 18) Vaginal delivery (*n* = 24)	●●●○
Fenizia et al., 2020 [82] Italy	*n* = 31 Median age: 30	Case report	NR	Admission to ICU and invasive ventilation (*n* = 1)	Third trimester (weeks median: 39)	Vaginal delivery (*n* = 25) Caesarean section (*n* = 6)	●●○○
Ferraiolo et al., 2020 [81] Italy	*n* = 1 Age: 30	Case report	Asymptomatic	Asymptomatic	Third trimester (38 weeks gestational age)	Urgent cesarean section	●○○○
Fontanella et al., 2020 [34] the Netherlands and Ireland	*n* = 2 Age: 39 years and 29 years	Case report	Fever ≥37.3 °C (*n* = 2) (1) Cough and increasing dyspnea, thoracic pain with deep breathing (2) Productive cough, sore throat, and diarrhea	NR	Third trimester (31 weeks gestational age) (40 weeks gestational age)	Cesarean (*n* = 2)	●○○○
Gabriel et al., 2020 [80] Spain	*n* = 7 Median age: 33–43	Observational prospective	Asymptomatic (*n* = 6) Fever, malaise, myalgia, headache (*n* = 1)	NR	Third trimester (≥38 weeks gestational age)	Vaginal delivery (*n* = 6) Cesarean section (*n* = 1)	●○○○
Gidlöf et al., 2020 [35] Sweden	*n* = 1 Age: 34 years	Case report	Hoarseness Increasing malaise Severe headache and photophobia	Oxygen saturation dropped to 87% Pulmonary edema/embolism	Third trimester (36 weeks gestational age)	Emergency cesarean—dichorionic twin pregnancy (*n* = 2)	●○○○
Hantoushzadeh et al., 2020 [36] Iran	*n* = 9 Age: 25 to 49 years	Case series	Fever (*n* = 9) Cough (*n* = 9) Dyspnea (*n* = 6) Myalgia (*n* = 4)	NR	Second trimester (*n* = 2) Third trimester (*n* = 7)	Cesarean delivery (*n* = 6) Vaginal delivery (*n* = 1)	●●○○
Hosier et al., 2020 [79] USA	*n* = 1 Age: 35	Case report	Fever, malaise, nonproductive cough, diffuse myalgias, anorexia, nausea, and diarrhea	NR	Second trimester (22 weeks gestational age)	Urgent cesarean section	●○○○
Iqbal et al., 2020 [37] USA	*n* = 1 Age: 34 years	Case report	Fever, chills, dry cough, and myalgia	Fever 38.5 °C	Third trimester (39 weeks of gestation)	Spontaneous vaginal delivery (*n* = 1)	●○○○
Kalafat et al., 2020 [38] Turkey	*n* = 1 Age: 32 years	Case report	Nausea Dyspnea Swollen left leg	NR	Third trimester (35 weeks gestational age)	Cesarean (*n* = 1)	●○○○
Karami et al., 2020 [39] Iran	*n* = 1 Age: 27 years	Case report	Fever, cough, and myalgia for 3 days	NR	Third trimester (30 and 3/7 weeks gestation)	Vaginal delivery (*n* = 1)	●○○○
Khan et al., 2020 [5] China	*n* = 3 Age: 27 to 33 years	Case report	Cough (*n* = 3) Fever (≥37.3 °C) (*n* = 2) Chest tightness (*n* = 1)	NR	Third trimester (≥34 weeks of gestation)	Vaginal delivery (*n* = 3)	●●○○
Kirtsman et al., 2020 [40] Canada	*n* = 1 Age: 40 years	Case report	Cough Pyrexia Tachycardic (110–121 beats/min) Fever (39 °C)	NR	Third trimester (35 + 3 weeks gestation)	Cesarean delivery (*n* = 1)	●●○○
Knight et al., 2020 [41] UK	*n* = 427	Cohort study	Fever (*n* = 280) Cough (*n* = 240) Breathlessness (*n* = 160) Tiredness or lethargy (*n* = 70) Headache (*n* = 60)	NR	Second trimester (≤26 weeks) (*n* = 4) Third trimester (>27 weeks) (*n* = 96)	Caesarean, maternal indication due to SARS-CoV-2 (*n* = 42) Caesarean, other indication (*n* = 114) Operative vaginal (*n* = 28) Unassisted vaginal (*n* = 78)	●●●●
Lee et al., 2020 [7] Korea	*n* = 1 Age: 35 years	Case report	Fever (>38 °C), mild sore throat and cough	NR	Third trimester (37 weeks gestational age)	Cesarean delivery (*n* = 1)	●○○○
Li et al., 2020 [43] China	*n* = 16 Age: 26 to 37 years	Case-control study	Fever (*n* = 4) Cough (*n* = 4)	Fever (*n* = 8)	Third trimester (Mean weeks gestational age = 38 ± 0.2)	Total: 17 babies Vaginal delivery (*n* = 2) Cesarean section (*n* = 14) Twin pregnancy	●●○○
Li et al., 2020 [44] China	*n* = 1 Age: 30 years	Case report	Fever (37.2 °C), chills, or shortness of breath	NR	Third trimester (35 weeks gestational age)	Emergency cesarean section (*n* = 1)	●○○○
Li et al., 2020 [42] China	*n* = 1 Age: 31 years	Case report	Fever and dyspnea to acute respiratory distress syndrome and septic shock	NR	Third trimester (35 + 2 weeks gestational age)	Cesarean delivery (*n* = 1)	●○○○
Liao et al., 2020 [45] China	*n* = 1 Age: 25 years	Clinical case reported in a letter to the editor	Fatigue and mild dry cough for 3 days Fever (38.3 °C)	NR	Third trimester (35 weeks gestational age)	Emergency cesarean section (*n* = 1)	●○○○
Liu et al., 2020 [9] China	*n* = 16 Age: 22–42 years	Retrospective study	Fever (*n* = 7) Cough (*n* = 6) Short of breath (*n* = 2) Fatigue (*n* = 3)	Fever (*n* = 5)	Third trimester (>22 weeks gestational age)	Cesarean section (*n* = 10)	●●●○
Liu et al., 2020 [46] China	*n* = 15 Age: 23 to 40 years	Case report	Fever (*n* = 13) Cough (*n* = 9) Sore throat (*n* = 1) Dyspnea (*n* = 1) Myalgia (*n* = 3) Fatigue (*n* = 4) Diarrhea (*n* = 1) Asymptomatic (*n* = 2)	Fever (*n* = 1)	First and third trimesters (≥27 weeks gestational age) (12 to 38 weeks)	Cesarean section (*n* = 10) Vaginal delivery (*n* = 1)	●●○○
Liu et al., 2020 [47] China	*n* = 3 Age: 30 to 34 years	Case series study	Fever (37.8 °C) (*n* = 2) Cough (*n* = 2)	NR	Third trimester (>38 weeks gestational age)	Cesarean section (*n* = 2) Natural childbirth (*n* = 1)	●●○○
Lv et al., 2020 [78] China	*n* = 1 Age: 28	Case report	Fever and cough	NR	Third trimester (31 weeks gestational age)	Cesarean section	●○○○
Lokken et al., 2020 [48] USA	*n* = 46 Age: 26 to 34 years	Retrospective study	Symptomatic (*n* = 43) Cough (*n* = 30) Fever or chill (*n* = 22) Nasal congestion (*n* = 21) Shortness of breath/dyspnea (*n* = 19) (44.2%) Asymptomatic (*n* = 3)	NR	Second or third trimester	Vaginal (*n* = 5) Cesarean (*n* = 3) Preterm birth at 33 weeks (*n* = 1)	●●●○
London et al., 2020 [49] USA	*n* = 68 Age: ≥30 years	Retrospective cohort study	Fever (*n* = 46) Cough (*n* = 46) Shortness of breath (*n* = 46) Sore throat (*n* = 46) Nausea (*n* = 46) Vomiting (*n* = 46) Asymptomatic (*n* = 22)	NR	First trimester (17 weeks gestational age) (*n* = 1) Second trimester (25 and 26 weeks) (*n* = 2) Third trimester (*n* = 65)	Cesarean delivery (*n* = 22)	●●●○
Lowe et al., 2020 [71] Australia	*n* = 1 Age: 31 years	Case report	Initially remained asymptomatic Fever	NR	Third trimester (40 weeks gestational age)	Vaginal delivery	●○○○
Martínez-Perez et al., 2020 [50] Spain	*n* = 82 Age: 33 years	Cohort	Symptomatic gestates (*n* = 60) Asymptomatic pregnant women (*n* = 22)	NR	Third trimester (≥29 weeks gestational age)	Delivered vaginally (*n* = 49) Cesarean delivery (*n* = 33)	●●●○
Patanè et al., 2020 [51] Italy	*n* = 22 Age: 33 years	Retrospective cohort study	Fever 38 °C (*n* = 2) Dry cough (*n* = 2)	NR	Third trimester (≥37.6 weeks gestational age)	Vaginal delivery (*n* = 1) Cesarean delivery (*n* = 1)	●●○○
Penfield et al., 2020 [52] USA	*n* = 32 Age: 22 to 40 years	Retrospective cohort study	NR	NR	Second and Third trimester (≥26 weeks)	Cesarean delivery (*n* = 4) Normal spontaneous vaginal delivery (*n* = 7)	●●●○
Peng et al., 2020 [53] China	*n* = 1 Age: 22 to 40 years	Case report	Fever, fatigue, shortness of breath	NR	Third trimester (35.2 weeks gestational age)	Cesarean (*n* = 1)	●○○○
Pereira et al., 2020 [77] Spain	*n* = 60 Median age: 34 years	Descriptive	Fever and cough	Admission to ICU (*n* = 1) (with HELLP syndrome)	Third trimester (median: 32 weeks)	During the study period, 23 women delivered: Cesarean section (*n* = 5) Vaginal (*n* = 18)	●○○○
Qiancheng et al., 2020 [54] China	*n* = 28 Mean maternal age 30 26.75–32	Single-center, retrospective study	Cough (*n* = 7) Fever (*n* = 5) Abdominal pain (*n* = 5) Dyspnea (*n* = 2) Malaise (*n* = 1)	NR	First trimester (*n* = 3) Second trimester (*n* = 1) Third trimester (*n* = 24)	Cesarean section (*n* = 17) Vaginal delivery (*n* = 5)	●●○○
Savasi et al., 2020 [55] Italy	*n* = 91 Age: 15 to 48 years	Retrospective cohort study	Fever (*n* = 54) Cough (*n* = 62) Dyspnea (*n* = 27)	Symptomatic (*n* = 10)	First trimester (*n* = 4) Second trimester (*n* = 13) Third trimester (*n* = 50)	Cesarean (*n* = 31) Vaginal (*n* = 36)	●●●○
Schwartz et al., 2020 [76] Iran	*n* = 9 Age: 28	Retrospective cohort study	NR	NR	Third trimester (>28 weeks gestational age)	Cesarean section (*n* = 8) Spontaneous vaginal delivery (*n* = 1)	●●○○
Sentilhes et al., 2020 [56] France	*n* = 38 Age: 19 to 42 years	Retrospective single-center study	Fatigue (*n* = 38) Cough (*n* = 25) Anosmia or ageusia (*n* = 18) Fever (*n* = 10)	NR	Third trimester (mean weeks gestational age 29.3 ± 8.5)	Cesarean (*n* = 6) Vaginal (*n* = 10)	●●○○
Sisman et al., 2020 [75] USA	*n* = 1 Age: 37	Case report	Fever	NR	Third trimester (34 weeks gestational age)	Vaginal delivery (*n* = 1)	●○○○
Siying et al., 2020 [57] China	*n* = 1 Age: 33	Case report	Dry cough 1 day before admission, without sputum, sore throat, fatigue	Dry cough	Third trimester (37 weeks gestational age)	Emergency caesarean section (*n* = 1)	●○○○
Vivanti et al., 2020 [74] France	*n* = 1 Age: 23	Case report	Fever (38.6 °C), severe cough, and abundant expectoration	NR	Third trimester (35 + 2 weeks of gestation)	Cesarean delivery (*n* = 1)	●○○○
Wang et al., 2020 [59] China	*n* = 1 Age: 28	Case report	Fever	NR	Third trimester (30 weeks pregnant)	Emergency cesarean (*n* = 1)	●○○○
Wang et al., 2020 [58] China	*n* = 1 Age: 34	Case report	Fever (37.8 ℃)	NR	Third quarter (40 weeks gestational age)	Emergency cesarean (*n* = 1)	●○○○
Wu et al., 2020 [60] China	*n* = 23 Age: 21–37 years	Case report	Cough (*n* = 6), Fever (*n* = 4) Nasal congestion (*n* = 1) Clinically asymptomatic (*n* = 15)	NR	First trimester (*n* = 3) (≤12 weeks) Third trimester (*n* = 20) (≥28 weeks gestational age)	Cesarean section (*n* = 18) Vaginal delivery (*n* = 2)	●●○○
Wu et al., 2020 [61] China	*n* = 13 Age: 26–40 years	Descriptive study, retrospective	Fever (*n* = 8) Cough (*n* = 5) Dyspnea (*n* = 1) Myalgia (*n* = 1) Diarrhea (*n* = 1)	NR	First trimester (*n* = 5) Second trimester (*n* = 3) Third trimester (*n* = 5)	Caesarean section (*n* = 4) Natural delivery (*n* = 1)	●●○○
Xiong et al., 2020 [62] China	*n* = 1 Age: 25 years	Case report	Fever (38 °C) Dry cough Shivering	NR	Third trimester (33 weeks gestational age)	Vaginal delivery six hours after (*n* = 1)	●●○○
Yan et al., 2020 [63] China	*n* = 116 Mean maternal age 30.8 ± 3.8	Descriptive study, retrospective	Fever (*n* = 59) Cough (*n* = 33) Clinically asymptomatic (*n* = 27)	NR	Third trimester (≥ 38 weeks gestational age)	Cesarean delivery (*n* = 85) Vaginal delivery (*n* = 14)	●●●○
Yang et al., 2020 [64] China	*n* = 26 Age: 21 to 40 years	Retrospective study	Fever (13 cases), cough (10 cases), vomiting (1 case)	NR	Third trimester (≥30 weeks gestational age)	Cesarean section (*n* = 20); Vaginal (*n* = 6 cases)	●●○○
Yang P et al., 2020 [65] China	*n* = 7 Age: 21 to 40 years	Case report	Fever (*n* = 5) Cough (*n* = 1) Abdominal pain (*n* = 1)	Fever	Third trimester (≥36 weeks gestational age)	Cesarean delivery (*n* = 7)	●●○○
Yu et al., 2020 [66] China	*n* = 7 Age: 29 to 34 years	Descriptive study, retrospective	Fever (*n* = 6) Cough (*n* = 1) Shortness of breath (*n* = 1) Diarrhea (*n* = 1)	NR	Third trimester (≥37 weeks gestational age)	Cesarean delivery (*n* = 7)	●●○○
Yu et al., 2020 [67] China	*n* = 1 Age: 35	Descriptive study, retrospective	Low fever and dry cough	Dyspnea and cyanosis	Third trimester (34 weeks gestational age)	Vaginal delivery (*n* = 1)	●○○○
Yue et al., 2020 [73] China	*n* = 14 Mean maternal age 30.1 ± 3.4	Case series study	Fever (*n* = 4)	NR	Third trimester (mean weeks gestational age 38 ± 0.4)	Emergency cesarean (*n* = 13)	●●○○
Zeng et al., 2020 [68] China	*n* = 33 Age: 24 to 34 years	Case report	Cough (*n* = 10) Fever on admission (*n* = 8)	Fever (*n* = 5)	Third trimester (≥31 weeks gestational age)	Cesarean delivery because of meconium-stained amniotic fluid Cesarean delivery (*n* = 26) Natural childbirth (*n* = 7)	●●●○
Zhang et al., 2020 [69] China	*n* = 16 Age: 24 to 34 years	Retrospective study	Cough (*n* = 3) Chest tightness (*n* = 3) Shortness of breath (*n* = 3) Diarrhea (*n* = 3)	NR	Third trimester (≥38 weeks gestational age)	Cesarean section (*n* = 10)	●●○○
Zhu et al., 2020 [70] China	*n* = 9 Mother of the twins (*n* = 1) Age: 25 to 34 years	Retrospective study	Fever (*n* = 7) Cough (*n* = 4) Diarrhea (*n* = 1)	NR	Third trimester (≥31 weeks gestational age)	Cesarean section (*n* = 7) Vaginal delivery (*n* = 2)	●●○○

NR: Not reported; * Quality of evidence based on GRADE classification in four categories: Very low quality, low quality, moderate quality, or high quality.

**Table 2:** Comorbidities and complications in pregnant women diagnosed with COVID-19.

Gestational Age	Comorbidity	Complications
Second trimester	Psoriasis [79] Severe hypertension [79] Coagulopathy [79]	Preeclampsia [79]
Third trimester	Asthma [21,23,50,56] Chronic comorbidity [82] Chronic hypertension [22,23] Type 2 diabetes mellitus [18,21,22,23,26,75] Dysfunction of blood coagulation [64] Hepatitis B [9,43] History of frequent bacterial infections (sinusitis, skin infection, and bronchitis) during this pregnancy [40] Hypertension [26,35,41,43,63] Hypothyroidism [59,64,66] Polycystic ovary syndrome [66] Mild-intermittent asthma [22] Obesity [21,22,34,35,50,56,69,75] Obstructive sleep apnea [21] Polycystic ovary syndrome [43]	Abnormal placenta (placenta previa) [70] Complete prior placenta [27] Complications in pregnancy ([73] Gestational diabetes [9,21,28,33,34,35,40,50,56,63,64,69] Gestational hypertension [9,21,25,56,64] Pneumonia secondary to COVID-19 [85] Sepsis [85] Maternal COVID-19 pneumonia [68] Intrauterine fetal distress [73] Placental detachment [27] Preeclampsia [25,28,50,63,69] Previous placenta [30] Severe preeclampsia [64]
Second and third trimesters *	Asthma [41,48] Cardiac disease [41] Diabetes [41] Hypertension [41] Obese [41] Overweight or obese [48] Type 2 diabetes [48]	Gestational diabetes [41,48] Gestational hypertension [48]
First, second, and third trimesters *	Asthma [49] Autoimmune disease [55] Chronic hepatitis B virus infection [54] Chronic hypertension [49] Diabetes [49,54] Endocrine disease [55] Hypertension [54] Hypothyroidism [54] Metabolic diseases [55] Obesity [55]	Gestational diabetes [49] Preeclampsia [49]

* There was no stratification of comorbidity and complications for the gestational semester.

**Table 3:** Results for newborn placentas and breast milk that tested positive for SARS-CoV-2 after birth.

First Author and Year	Diagnosis Test	Type of Delivery	Positive COVID-19 Test Results
**NEWBORNS**			
Alzamora et al., 2020 [18]	Nasopharyngeal swab was obtained for SARS-CoV-2 RT-PCR	Emergency cesarean section (*n* = 1)	Nasopharyngeal swab, 16 h after delivery, was positive for SARS-CoV-2 RT-PCR, and immunoglobulin (Ig)-M and IgG for SARS-CoV-2: Negative
Buonsenso et al., 2020 [24]	RT-PCR	Cesarean section (*n* = 1)	SARS-CoV-2 positive at 15 days of life, although asymptomatic
Facchetti et al., 2020 [83]	SARS-CoV-2 RNA on nasopharyngeal swab	Vaginal delivery was induced (*n* = 1)	Resulted inconclusive (amplification of less than three genes), while it was positive 36 and 72 h after birth and at the age of 17 days
Fenizia et al., 2020 [82]	Nasopharyngeal newborn swab was obtained for SARS-CoV-2 RT-PCR after the baby was cleaned	Vaginal delivery (*n* = 25) Caesarean section (*n* = 6)	Viral RNA positive in newborns (*n* = 2)
Ferrazzi et al., 2020 [33]	RT-PCR	(1) Elective cesarean section (*n* = 2) (2) Vaginal delivery (*n* = 1)	(1) Newborns had a positive test for COVID-19 infection at days one and three, respectively (2) The first test for SARS-CoV-2 was equivocal a few hours after delivery, but positive three days later
Hantoushzadeh et al., 2020 [36]	SARS-CoV-2 NAT	Cesarean section (*n* = 1)	Negative on day 1 of life but converted to positive on day 7 of life with an accompanying lymphopenia (nadir white blood cell 8.9, with 26% lymphocytes) The neonate was intubated for prematurity, developed pneumonia at day of life 2 There was maternal death
Knight et al., 2020 [41]	Detection of viral RNA on polymerase chain reaction testing of blood or a nasopharyngeal swab or aspirate	Cesarean section (*n* = 4) Vaginal birth (*n* = 2)	Positive test <12 h of age (*n* = 6) Positive test ≥12 h of age 6 (*n* = 6)
Kirtsman et al., 2020 [40]	RT-PCR Placental swabs (both maternal and fetal sides) were obtained Placental tissue was sent for PCR and histopathologic examination Nasopharyngeal swabs were obtained from the neonate on the day of birth, day 2, and day 7, after thorough cleansing of the baby and before contact with the mother	Cesarean section (*n* = 1)	All 3 of the neonate’s nasopharyngeal swabs were positive for SARS-CoV-2 gene targets via RT-PCR testing; neonatal plasma tested positive on day 4, and stool was positive on day 7
Martínez-Perez et al., 2020 [50]	SARS-CoV-2 RNA RT-PCR	Vaginal delivery (*n* = 2) Cesarean section (*n* = 1)	Three newborns tested within 6 h after birth had a positive SARS-CoV-2 RT-PCR result Repeat testing at 48 h was negative None developed COVID-19 symptoms within 10 days
Patanè et al., 2020 [51]	SARS-CoV-2 RNA RT-PCR	Vaginal delivery (*n* = 1) Cesarean section (*n* = 1)	(1) The newborn had positive NP swabs immediately at birth, after 24 h, and after 7 days; he remained asymptomatic (2) Neonatal NP swab was 60 negative at birth and turned positive on day 7, with no contact between mother and neonate during that period
Savasi et al., 2020 [55]	Pharyngeal swab sampled for SARS-CoV-2	Vaginal delivery (*n* = 3) Cesarean section (*n* = 1)	Positive on the seventh day (*n* = 1)
Schwartz et al., 2020 [76]	Infant RT-PCR testing	Cesarean section (*n* = 8) Spontaneous vaginal delivery (*n* = 1)	Positive by RT-PCR for SARS-CoV-2 (*n* = 9): 1 h after delivery (*n* = 1) 2 h after delivery (*n* = 1) DOL 2 (*n* = 1) DOL 3 (*n* = 1) DOL 4 (*n* = 2) DOL 6 (*n* = 1) DOL 7 (*n* = 1) DOL 24 (*n* = 1)
Sisman et al., 2020 [75]	Nasopharyngeal swab by RT-PCR for SARS-CoV-2 at 24 and 48 h of life	Vaginal delivery (*n* = 1)	Positive by nasopharyngeal swab
Vivanti et al., 2020 [74]	Nasopharyngeal and rectal swabs were first collected after having cleaned the baby at 1 h of life, and they were tested with RT-PCR Blood and nonbronchoscopic bronchoalveolar lavage fluid were collected for RT-PCR	Cesarean delivery (*n* = 1)	Nasopharyngeal and rectal Blood and nonbronchoscopic bronchoalveolar lavage fluid Clear amniotic fluid was collected prior to rupture of membranes during cesarean section and tested positive for both the E and S genes of SARS-CoV-2
Zeng et al., 2020 [68]	SARS-CoV-2 real-time reverse transcriptase–polymerase	Cesarean section (*n* = 3)	(1) Nasopharyngeal and anal swabs were positive for SARS-CoV-2 on days 2 and 4 of life (2) Nasopharyngeal and anal swabs were positive for SARS-CoV-2 on days 2 and 4 of life and negative on day 6 (3) Nasopharyngeal and anal swabs were positive for SARS-CoV-2 on days 2 and 4 of life and negative on day 7
Wang et al., 2020 [65]	SARS-CoV-2 RNA RT-PCR Pharyngeal swab	Emergency cesarean section (*n* = 1)	The result of pharyngeal swab for SARS-CoV-2 was positive at 36 h after birth
Yu et al., 2020 [67]	RT-PCR for SARS-CoV-2	Cesarean section (*n* = 1)	Nucleic acid test for the throat swab of one neonate (child of patient 1) was positive at 36 h after birth
**IgG Antibody**			
Dong et al., 2020 [31]	CT and RT-PCR nasopharyngeal swabs; and IgM and IgG antibody, cytokine, and other biochemistry tests in blood Vaginal secretions	Cesarean section (*n* = 1)	A neonate born to a mother with COVID-19 had elevated antibody levels (IgM) and abnormal cytokine test results 2 h after birth Nasopharyngeal swabs taken from 2 h to 16 days of age were negative Mother’s breast milk had a negative RT-PCR test result
Fenizia et al., 2020 [82]	Umbilical cord plasma: SARS-CoV-2 RT-PCR and SARS-CoV-2 IgG and IgM chemiluminescence immunoassay	Vaginal delivery (*n* = 25) Caesarean section (*n* = 6)	Viral RNA positive + IgG positive in umbilical cord plasma (*n* = 1) IgG positive in umbilical cord plasma (*n* = 10) IgM positive + IgG positive in umbilical cord plasma (*n* = 1)
**Umbilical Cord**			
Fenizia et al., 2020 [82]	Umbilical cord: SARS-CoV-2 RT-PCR	Vaginal delivery (*n* = 25) Caesarean section (*n* = 6)	Viral RNA positive in umbilical cord (*n* = 1)
**Vaginal Swab**			
Fenizia et al., 2020 [82]	Vaginal swab: SARS-CoV-2 RT-PCR	Vaginal delivery (*n* = 25) Caesarean section (*n* = 6)	Viral RNA positive in vaginal swab (*n* = 1)
Yang et al., 2020 [64]	RT-PCR for SARS-CoV-2 SARS-CoV-2IgM/IgG antibodies rapid test kit	Cesarean section (*n* = 1) Premature rupture of fetal membranes	Elevated IgM level of SARS-CoV-2 2 h after her birth Testing on her nasopharyngeal swab was negative (tested twice)
**Symptomatic Cases**			
Fan et al., 2020 [32]	RT-PCR nasopharyngeal swab, maternal serum, placental tissues, umbilical cord blood, amniotic fluid, vaginal swabs, and breast milk	Cesarean section (*n* = 2)	Symptoms, suspected case: Two babies showed symptoms but failed to detect SARS-CoV-2 in any of the samples, including the newborn’s nasopharyngeal swab, maternal serum, placental tissues, umbilical cord blood, amniotic fluid, vaginal swabs, and breast milk
Gidlöf et al., 2020 [35]	RT-PCR	Emergency cesarean section (*n* = 2)	Twins symptoms, suspected cases: At 22 min after delivery developed breathing problems On the second day, she had a cyanotic attack while feeding Both twins had negative nasopharyngeal COVID-19 tests taken at 34 h and 4½ days of age COVID-19 tests performed on breast milk and maternal vaginal secretion on the fifth day were also negative
**Placenta**			
Algarroba et al., 2020	Electron microscopy	Cesarean delivery	A single virion was visible invading a syncytiotrophoblast A single virion was also visualized in a microvillus
Baud et al., 2020 [20]	Maternal—RT-PCR Deep nasopharyngeal Deep nasopharyngeal control Vagina Blood Fetus—RT-PCR Umbilical cord blood Amniotic fluid negative sterile Fetal armpit Placental submembrane Placental cotyledon Fetal anus Fetal liver Fetal thymus Fetal lung	Vaginal delivery (*n* = 1)	Placental submembrane—positive sterile Placental cotyledon—positive
Facchetti et al., 2020 [83]	Histological, immunohistochemical, in situ SARS-CoV-2 RNA, RNA in situ hybridization and electron microscopy	Vaginal delivery was induced (*n* = 1)—third trimester	Placenta: Tested positive for SARS-CoV-2, after detecting the presence of S-protein-specific transcripts by RNA-in situ hybridization; immunostains for SARS-CoV-2 proteins showed positivity in the cytoplasm of the syncytiotrophoblast for both S-protein and *n*-protein
Fenizia et al., 2020 [82]	SARS-CoV-2 RT-PCR	Vaginal delivery (*n* = 25) Caesarean section (*n* = 6) Third trimester	Viral RNA positive in placenta (*n* = 2)
Ferraiolo et al., 2020	SARS-CoV-2 RT-PCR	Urgent cesarean section (*n* = 1)—third trimester	The definitive histological analysis of the placenta did not describe substantial macroscopic alterations, except for mild subchorionic deposition of fibrin and for the presence of a single ischemic area in the thickness of the chorionic disc
Hosier et al., 2020	SARS-CoV-2 RT-PCR Whole-genome sequenced Histological examination Electron microscopy	Urgent cesarean section (*n* = 1)—second trimester	Placenta and umbilical cord were positive for SARS On histological examination, SARS-CoV-2 localized predominantly to the syncytiotrophoblast cells of the placenta Analysis of the placental region adjacent to the umbilical cord identified virus particles within the cytosol of placental cells consistent with the size and appearance of SARS-CoV-2
Penfield et al., 2020 [52]	SARS-CoV-2 RNA RT-PCR	Cesarean section (*n* = 3)	Infected placentas (*n* = 1) or membrane swabs (*n* = 2)
Patanè et al., 2020 [51]	SARS-CoV-2 RNA RT-PCR	Vaginal delivery (*n* = 1) Cesarean section (*n* = 1)	SARS-CoV-2 RNA in the placentas
Vivanti et al., 2020 [74]	SARS-CoV-2 RNA RT-PCR	Cesarean delivery (*n* = 1) Third trimester	Placental infection as positive for SARS-CoV-2 RNA
**Breast Milk**			
Costa et al., 2020 [84]	SARS-CoV-2 RT-PCR in six breast milk samples	Cesarean delivery (*n* = 2)—third trimester	Three of six breast milk samples (50%) had a cycle threshold value <40 (the value interpreted as positive for SARS-CoV-2 RNA), indicating that patient 1 excreted virus in her breast milk, albeit intermittently (*n* = 1)
Fenizia et al., 2020 [82]	Five days after delivery (T2), transitional/mature breast milk samples were collected from all breastfeeding women Diagnosis test SARS-CoV-2 RT-PCR and SARS-CoV-2 IgG and IgM chemiluminescence immunoassay	Vaginal delivery (*n* = 25) Caesarean section (*n* = 6) Third trimester	Viral RNA positive and IgM positive in breast milk (*n* = 1)
Wu et al., 2020 [61]	SARS-CoV-2 RNA RT-PCR—1st day after delivery Breast milk samples from three women were collected on the 1st, 6th, and 27th days after delivery Vaginal swabs Neonatal throat and anal swabs were collected on the 1st and 3rd days after birth	Caesarean section (*n* = 4) Natural delivery (*n* = 1)	Detection of SARS-CoV-2 in breast milk

RT-PCR: Reverse transcriptase—polymerase chain reaction; SARS-CoV-2: Severe acute respiratory syndrome coronavirus 2; DOL: Days of life.
